# Opioid overdose crises during the COVID-19 pandemic: implication of health disparities

**DOI:** 10.1186/s12954-021-00534-z

**Published:** 2021-08-16

**Authors:** Ishika Patel, Lauren A. Walter, Li Li

**Affiliations:** 1 Department of Psychiatry and Behavioral Neurobiology, University of Alabama At Birmingham, 1720 University Blvd., Birmingham, AL 35294 USA; 2 Department of Emergency Medicine, University of Alabama At Birmingham, Birmingham, AL USA

**Keywords:** Opioid, Overdose, Death, Racial disparity, Singlehood

## Abstract

**Background:**

Recent data suggest a disproportionate impact of opioid overdoses on Black Americans. The study aims to describe emergency department (ED) visits at a Southern, urban ED pertaining to opioid overdose and associated health disparities.

**Methods:**

Patients presenting to the ED at the University of Alabama at Birmingham Hospital with opioid overdoses from January 1 to October 31, 2019, and from January 1 to October 31, 2020, were identified from electronic medical records.

**Results:**

The total number of opioid overdose visits increased 9.7% (556 to 611) between January and October 2020 compared with 2019. Among patients who presented with opioid overdose, the mean ages were 50.3 years and 48.3 years, in 2019 and 2020, respectively. In both 2019 and 2020, more Blacks than whites were treated for opioid overdose in the ED (284 vs. 258 in 2019, and 306 vs. 271 in 2020) although 28 patients did not record their race in 2020. Consistently, more overdose deaths were observed in Blacks than in whites in 2020. More individuals seeking opioid overdose treatment were single in both years.

**Conclusions:**

The study reported a greater number of visits for opioid overdoses from January to October of 2020 in an ED of a southeastern region, as well as higher overdose deaths in Blacks. Our findings highlight the importance of substance use treatment, harm reduction, and overdose prevention efforts that should be immediately present to reduce opioid overdose, especially for vulnerable populations in the South, i.e., Black community, and individuals experiencing singlehood.

## Introduction

The drug overdose epidemic in the United States (US) continues to cause substantial morbidity and mortality. Overdose cases have increased significantly following the start of the COVID-19 pandemic. Multiple causes have been attributed to increased overdose. Many individuals have become more vulnerable during the pandemic and were impacted by unprecedented stress, unstable home and community structures, and drug supply change which worsened their mental health and triggered those individuals to use or reuse and experience unintentional opioid overdose [1]. It is also important to understand that access to treatment programs was restricted during the pandemic and was often only made available to the most urgent cases. Additionally, the public was encouraged to remain socially distant and isolated in their homes, which caused many individuals who normally used drugs in groups to use alone, leaving them without a potential rescue [2]. Recently, national epidemiologic data have shown an acceleration in the number of overdose-related deaths from May 2019 to May 2020, the pinnacle of the COVID-19 pandemic [3]. In 12 months, more than 81,000 deaths arose from drug overdose-related consequences [3].

While general visits to Emergency Departments (EDs) transiently plunged during early to mid-pandemic, visits related to opioid misuse increased across the country [4]. In Kentucky alone, transportation to EDs due to opioid overdose increased by 17%; in addition, there was a concomitant [5]. Seventy-one percent increase in ambulance calls. However, many patients refused treatment or ED transfer. Another study conducted at Virginia Commonwealth University (VCU) showed the number of patients with a nonfatal opioid overdose increased from 102 to 227 from March through June 2019 to March through June 2020 [6]. Current data suggest that the exacerbation of the opioid epidemic by the COVID-19 pandemic may be disproportionately affecting Black Americans [7]. The same VCU study also demonstrated that from March through June 2019 and March through June 2020, the number of Black patients with unintentional opioid overdose rose from 64 to 181, while the number of white patients only rose from 29 to 32, indicative of the presence and intensification of racial/ethnic disparities in opioid overdose during the COVID-19 pandemic [6].

The state of Alabama has been particularly impacted by the opioid epidemic and overdose death. In 2018, approximately half of the 775 overdose deaths reported in Alabama involved opioids [8]. Furthermore, recent reports show that opioid-related overdose deaths have increased by 32.5% in just the first six months of 2020 in Jefferson County, the most populous county in Alabama. Compared with other counties in Alabama, Jefferson County has the highest rate of opioid use disorder and its associated complications [9]. In addition to racial and ethnic disparities, it is important to consider the impact of other social determinants of health, such as marital status, on opioid overdose as well. Thus, this study aims to describe the changes in ED visits related to opioid overdose and any associated demographic disparities, including race and marital status, with the aim to provide a more complete assessment of the impact of the COVID-19 pandemic on patients living with opioid misuse and opioid use disorder.

## Methods

This retrospective cross-sectional study was approved by the Institutional Review Board at the University of Alabama at Birmingham (UAB), and the need for informed consent was waived. Patients who presented and were treated for opioid overdoses in the ED at the UAB Hospital from January 1 to October 31, 2019, and from January 1 to October 31, 2020, were identified from electronic medical records (EMR) based on the following International Classifications of Disease (ICD) codes: ICD-10 T40.0 × – T40.6 × codes with the exception of T40.5 × code. Our EMR clearly indicated whether patients experienced a fatal opioid overdose death versus non-fatal. Thus, extracted data included both non-fatal opioid overdose and fatal opioid overdose. Data on opioid overdose fatalities in November and December of 2020 were not included for analyses, because the retrospective review was conducted in November of 2020. Manual medical record reviews to abstract patients’ data were conducted by two research team members independently (IP and LL), and discrepancies were checked against the EMR. Demographic characteristics, including age, sex, race/ethnicity, and marital status, were also obtained from the EMR. We analyzed data from January through October of 2019 and 2020 and compared the number of total patients with opioid overdoses in these 2 years. Descriptive statistical analyses were performed using SPSS version 26 (IBM).

## Results

**Fig. 1:**
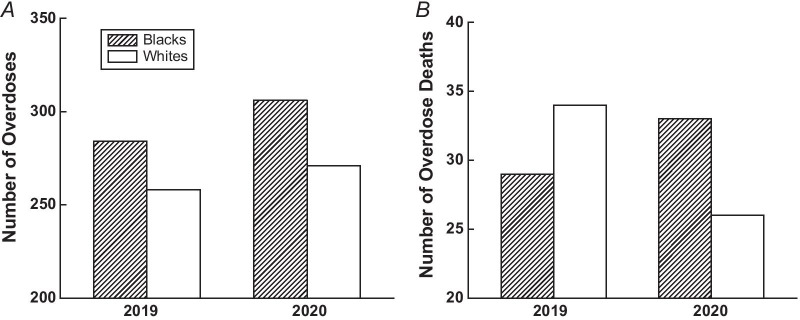
Comparisons between the number of overdoses in **a**, the number of overdose deaths in **b**, between Black patients and white patients

From January through October, there were 556 patients in 2019 and 611 in 2020 who presented to the ED for treatment of opioid overdose (Table 1). The mean (SD) ages of patients were 50.3 (15.9) years in 2019 and 48.3 (16.1) years in 2020. Among the 556 patients in 2019, 241 were females and 315 were males. Of the 611 patients in 2020, 274 were females, 309 were males, and 28 patients had not recorded their sex. In terms of race distribution among patients, there were more Black patients than whites who were treated in the ED for opioid overdose (Fig. 1a). As presented in Fig. 1a, 284 and 306 Black patients were treated for opioid overdose in 2019 and 2020, respectively. From January through October 2019, 63 patients died due to opioid overdose. This number slightly decreased to 59 in January through October in 2020 (Fig. 1b). However, among the 59 deaths from January through October in 2020, more Blacks (33 out of 59, or 56%) than whites (26 out of 59, or 44%) died due to an opioid overdose. None of 59 overdose deaths were from other races/ethnicities in 2020. In both 2019 and 2020, most patients seeking opioid overdose treatment were unmarried.

**Table 1:** The demographics of patients

	January–October 2019, *n* = 556	January–October 2020, *n* = 611
Age, mean (SD)	50.3 (15.9)	48.3 (16.1)
*Sex, N*		
Female	241	274
Male	315	309
Unknown	0	28
*Race, N*		
Blacks	284	306
Whites	258	271
Asians	13	6
Unknown	1	28
*Ethnicity, N*		
Non-Hispanic	531	558
Hispanic	5	1
Unknown	20	52
*Marital status, N*		
Single	315	309
Married	125	114
Divorced	60	80
Separated	6	16
Widowed	43	47
Unknown	7	45
*Religion, N*		
Yes	456	465
No/unknown	100	146

## Discussion

In this cross-sectional study, we have identified an approximately 9.7% increase in patients who were treated in the ED for opioid overdose from January to October 2020 compared to the same period in 2019. It was also observed that more Black patients than whites were treated in the ED for opioid overdose in both years. Furthermore, more Blacks than whites died of opioid overdose in 2020 in our sample. This reversal in the proportion of patients who were treated in the ED and who died by race is highly indicative of racial discrepancies that have intensified during the pandemic. In addition, findings support that individuals experiencing singlehood were also more likely to experience overdose. Our data indicate the presence of social disparities in this local sample within the southeastern region.

Increased opioid overdose deaths among Blacks have been reported recently by groups in Philadelphia and Virginia as well [10, 11]. In Philadelphia, more Black residents than white residents experienced higher rates of both fatal and non-fatal overdoses after the pandemic began [10]. These data suggest that racial disparities were intensified due to the pandemic. A similar finding was observed in an ED in Virginia; more Black patients made up a large percentage of the total non-fatal opioid overdose-related visits to the ED [6]. In an ED in Alabama, we observed more opioid overdoses visits in ED in 2020 overall, and more overdose deaths in Black patients specifically. Besides racial discrepancies, disparities are also present in those who are single. In our sample, more individuals experiencing overdose were single rather than married. It has been demonstrated previously that single status and social isolation are often related to the increased occurrence of diseases and mortality [12]. Social isolation and physical distancing, especially during the pandemic, may indicate poor mental health which may be one reason individuals resort to drug uses [12].

Many efforts have been initiated to combat the opioid epidemic and overdose rates; however, the epidemic seems to have been heavily impacted by the COVID pandemic, particularly among the vulnerable racial and social cohorts. There are several theories to explain this phenomenon, including social isolation which is anecdotally known to have caused an increased relapse rate for people who are in solid recovery [13]. Other factors include decreased access to medical assistance and treatment during the pandemic and a temporary interference in the distribution of naloxone, an opioid overdose reversal drug [14]. There has also been significant speculation that pandemic-related border closures interrupted the ‘usual’ heroin and drug supply entering the U.S., causing a ‘fundamental change in the pharmacology of the drug’ [15]. Due to this, what people are buying on the street may be cut with more fentanyl or other drugs. All of these factors may have contributed to increased opioid overdose cases, including non-fatal overdoses that were seen in our sample. Social need and social risk characteristics, including race and marital status, may result in particular vulnerability as the opioid epidemic and COVID-19 pandemic overlap.

Several limitations should be noted when interpreting these results. First, urine toxicology screening at UAB does not detect fentanyl, so we were not able to determine whether fentanyl specifically may have contributed to increased opioid overdoses. Secondly, we were unable to determine whether fatal overdoses were due to intentional or non-intentional behavior through the EMR data. Thirdly, urine toxicology screening in many patients showed polysubstance use, so it is difficult to determine the impact of other substances on our observed rates of overdoses or overdose deaths. Lastly, we are limited by our short observation period, i.e., 10 months, so further analysis with additional months of data to verify our findings is warranted.

## Conclusion

The COVID-19 pandemic has significantly emphasized the dangers of preexisting racial and social disparities and presents an opportunity for policymakers and health care workers to advocate for equal treatment and to expand treatment plans for proper and preventative care. In summary, our data provide further evidence of health disparities in the Black community and in individuals experiencing singlehood as it pertains to increased opioid overdose and overdose death that have been magnified during the COVID-19 pandemic. Our findings highlight the importance of substance use treatment, harm reduction, and overdose prevention efforts. Each should be immediately present to reduce opioid overdose, especially for the most vulnerable populations, including the Black community and individuals experiencing singlehood.

## Data Availability

The datasets used and/or analyzed during the current study are available from the corresponding author on reasonable request.

## Abbreviations

ED: Emergency Department
VCU: Virginia Commonwealth University
UAB: University of Alabama at Birmingham
EMR: Emergency Medical Records
ICD: International Classifications of Disease
SD: Standard deviation
